# Mechanisms and roles of the first stage of nodule formation in lepidopteran insects

**DOI:** 10.1093/jisesa/iead049

**Published:** 2023-07-05

**Authors:** Ryoichi Sato

**Affiliations:** Graduate School of Bio-Application and Systems Engineering, Tokyo University of Agriculture and Technology, Naka-cho 2-24-16, Koganei, Tokyo 184-8588, Japan

**Keywords:** nodule, hemocyte, insect immunity, cell mediated immunity, lepidopteran insect

## Abstract

Nodule formation is a process of cellular immunity in insects and other arthropods with open circulatory systems. Based on histological observations, nodule formation occurs in 2 stages. The first stage occurs immediately after microbial inoculation and includes aggregate formation by granulocytes. The second stage occurs approximately 2–6 h later and involves the attachment of plasmatocytes to melanized aggregates produced during the first stage. The first stage response is thought to play a major role in the rapid capture of invading microorganisms. However, little is known regarding how granulocytes in the hemolymph form aggregates, or how the first stage of the immunological response protects against invading microorganisms. Since the late 1990s, our understanding of the molecules and immune pathways that contribute to nodule formation has improved. The first stage of nodule formation involves a hemocyte-induced response that is triggered by pathogen-associated molecular pattern (PAMP) recognition proteins in the hemolymph regulated by a serine proteinase cascade and cytokine (Spätzle) and Toll signaling pathways. Hemocyte agglutination proceeds through stepwise release of biogenic amine, 5-HT, and eicosanoids that act downstream of the Toll pathway. The first stage of nodule formation is closely linked to melanization and antimicrobial peptide (AMP) production, which is critical for insect humoral immunity. Nodule formation in response to artificial inoculation with millions of microorganisms has long been studied. It has recently been suggested that this system is the original natural immune system, and enables insects to respond to a single invading microorganism in the hemocoel.

## Introduction

Nodule formation is a process that occurs in the cellular immunity system of insects and other arthropods with open circulatory systems (Ratcliffe and Rowley 1979). Efficient capture of microorganisms by nodules has been reported in *Pieris brassicae*, *Galleria mellonella*, *Clitumnus extradentatus*, *Schistocerca gregaria*, *Tenebrio molitor*, and *Dermatobia hominis*, implying that this system is common to a wide range of insects (Ratcliffe and Gagen 1977, Ratcliffe and Rowley 1979, Faraldo and Lello 2003). Histological observations, including by electron microscopy, were used to identify the basic mechanism of nodule formation in the 1970s, and revealed a two-stage process (Ratcliffe and Rowley 1979). The first stage occurs through the formation of aggregates, primarily by a granulocyte response immediately after microbial inoculation (Ratcliffe and Gagen 1977, Ratcliffe and Rowley 1979). The second stage occurs approximately 2–6 h later, when plasmatocytes attach to melanized aggregates produced during the first stage (Ratcliffe and Rowley 1979). The 2 stages of nodule formation are thought to have different functions, but only the functions of the first stage of nodule formation have been identified recently, as reported in this review.

Inoculation with an enormous number of microorganisms results in the formation of aggregates composed of granulocytes within 1 min in the first stage (Ratcliffe and Gagen 1977). In the silkworm (*Bombyx mori*), most microorganism-containing hemocyte aggregates form in the hemolymph within 30 s of injection with *Escherichia coli* cells (Arai et al. 2013). Therefore, the first stage was thought to play a major role in the response to, and rapid capture of, invading microorganisms (Ratcliffe and Gagen 1977). However, about the cellular and molecular mechanisms of aggregate formation facilitated by granulocytes and factors in the hemolymph, little has been addressed in previous reviews. In addition, a comprehensive understanding of the immunological functions involved in the first-stage response against invading microorganism was still needed.

However, studies have already began to elucidate the molecules and immune pathways that impact nodule formation in lepidopteran insects, since the late 1990s. Now, it has become clear that the first stage of nodule formation involves pathogen-associated molecular pattern (PAMP) recognition proteins in the hemolymph that regulate a signaling pathway consisting of the serine proteinase cascade and a cytokine (Spätzle). Hemocyte agglutination proceeds through the release of a biogenic amine, 5-HT, and eicosanoids, which function downstream via activation of the Toll pathway of granulocytes. It has also becoming clear that the first stage of nodule formation is closely linked to melanization and antimicrobial peptide (AMP) production, which are key responses in insect humoral immunity. While nodule formation has been investigated only in the context of the response to the invasion or inoculation of millions of microorganisms, it has been suggested that this system also acts on single invading microorganisms in the insect hemocoel (Otuka and Sato 2023).

Therefore, in this review, I provide a lot of compelling data and hypotheses regarding the regulatory mechanism of the first stage of nodule formation and its deep connections with other immune responses in lepidopteran insects to show that nodule formation is more than a simple reaction that aggregates granulocytes.

## Formation of Hemocyte Aggregates in the Hemolymph

Nodule-formation reactions have historically been studied by inoculating insects with a large number of bacteria or fungi, which facilitates observation. However, it is practically impossible for an insect to be invaded by millions of cells at once, which means that the experimental conditions that usually induce nodule formation do not occur in the natural world. This implies that the nodule formation reaction which is induced by such a condition is an artifact. To clarify the role of nodule formation in the cell-mediated immune response of insects, it is necessary to demonstrate that the response occurs even when only 1 (or a small number) of microorganisms invade. In addition, understanding the molecular mechanism of the first step at the cellular level also requires precise observation of the process of cell agglutination in the hemolymph.

**Fig. 1.**
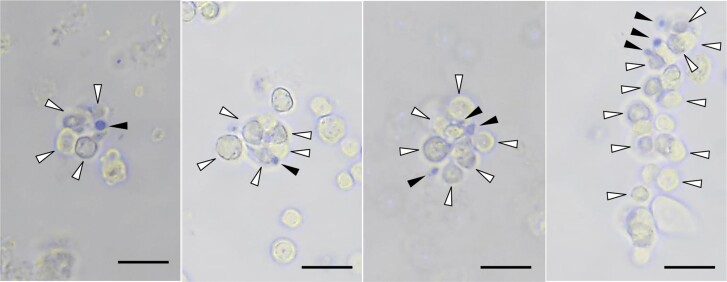
Photos of small *B. mori* larval hemocyte aggregates in hemolymph, induced by inoculation with *S. cerevisiae* cells. The photos were prepared for the manuscript by Otuka and Sato (2023), but were not published. Coomassie brilliant blue-stained *S. cerevisiae* cells (2.0 × 10^5^) were injected into the larvae. Hemolymph samples were collected after 1 min and observed with a phase-contrast inverted microscope. White and black arrowheads indicate hemocytes and *S. cerevisiae* cells, respectively. Scale bar = 20 μm. A, B) Hemocyte aggregation induced by a single *S. cerevisiae* cell. C, D) Hemocyte aggregates induced by a few *S. cerevisiae* cells.

Injection of *B. mori* larvae with yeast cells (2 × 10^5^) pre-stained with a protein staining blue dye, Coomassie Brilliant Blue resulted in the formation of approximately 500 hemocyte and yeast cell aggregates in the hemolymph within 1 min, with the number stabilizing thereafter (Otuka and Sato 2023). Observation of the aggregates 1 min after inoculation revealed small aggregates of 5–10 hemocytes and a few *Saccharomyces cerevisiae* cells. Many small aggregates contained only 1 *S. cerevisiae* cell (Fig. 1), implying that small aggregates can be induced by 1 or a small number of *S. cerevisiae* cells (Otuka and Sato 2023). Medium-sized aggregates contained 11–30 hemocytes and 2–10 *S. cerevisiae* cells, while large aggregates were composed of >31 hemocytes and numerous *S. cerevisiae* cells. The formation of larger aggregates was thought to depend on the artificial conditions produced by the injection of 2 × 10^5^*S. cerevisiae* cells. Hemocytes were consumed after inoculation with microbial cells for clearance from the hemolymph in *G. mellonella, P. brassicae*, and *B. mori* (Gagen and Ratcliffe 1976, Otuka and Sato 2023 ). These findings imply that the first stage of nodule formation involves the acute formation of aggregates composed of hemocytes and microbial cells invading the hemolymph (Otuka and Sato 2023).

Stanley et al. (2009) reached a similar conclusion to that described above after observing microaggregates in the hemolymph of *Manduca sexta* larvae 1 h after *S. marcescens* injection. Most of the aggregates in the hemolymph of *B. mori* were smaller than the nodules seen on organs 1 h after inoculation. These results may imply that the aggregates repeatedly coalesced with each other to generate larger aggregates that precipitated on the organs through the hemolymph to form nodules (Otuka and Sato 2023 , Ratcliffe and Gagen 1977). Therefore, the large nodules attached to organs that are visible with the naked eye may be an artifact created by the injection of large numbers of microbial cells. A more natural scenario involves invasion of the hemocoel by only a few microbial cells, leading to the formation of aggregates containing 5–10 hemocytes and 1–3 microbial cells (Fig. 1). Experiments that use a single or only a few microbial cells during inoculation should be performed to confirm the first stage of nodule formation.

Although not clearly visible in the photographs from these early studies, Ratcliffe and Gagen (1977) and Ratcliffe and Rowley (1979) described granulocytes undergoing degranulation and the release of an adhesive flocculent material from the hemocytes of *Galleria mellonella* that was in contact with bacteria. Immunohistochemical analysis has indicated that the aggregates from *B. mori* larval hemolymph are hemocytes tethered by a viscous protein, hemocytin which is an exocytosed substance from granulocytes (Arai et al. 2013, Otuka and Sato 2023). These results imply that the formation of small aggregates in the hemolymph of lepidopteran insects is due to the viscosity of a hemocytin orthologs that are homologs of mammalian von Willebrand factor released by the degranulation of granulocytes. As mentioned in Sections 2–4, degranulation of hemocytin-containing granules from granulocytes cannot be directly induced by attachment to microbial cells. Degranulation of hemocytin-containing granules appears to occur as a stepwise reaction consisting of PAMP recognition by PAMP recognition receptors (PRRs; BmLBP and BmMBP), activation of the hemolymph proteinase (BmHP8) and Spätzle1 (BmSPZ1) signaling pathways, granulocyte activation through Toll (BmToll10-3), and granulocyte activation by 5-HT and eicosanoids.

Phagocytosis was not observed during the first stage of nodule formation in many insect species inoculated with numerous organismal cells (Ratcliffe and Rowley 1979). Ratcliffe and Rowley (1979) summarized a common conclusion in many book chapters as follows: “Following the injection of test particles, such as carmine, polystyrene beads, India ink, erythrocytes, bacteria, fungi, yeast cell walls, mycetomes, and protozoa into the hemocoel, phagocytosis took place within 1 h”. Most of the microbial cells involved in the inoculation were captured by hemocytes as aggregates within 1 min (Otuka and Sato 2023). In addition, microbial cells were buried in the matrix containing hemocytin in the aggregate (Arai et al. 2013). These findings imply that hemocytes do not have the opportunity to attack microbial cells individually to induce phagocytosis because of the acute aggregation and matrix coating.

The initial response of innate immunity of insects is now thought to involve a cell-mediated aggregation immune reaction called nodule formation, which may play a more important role than phagocytosis. However, bacterial cells not cleared by nodule formation after inoculation of *B. mori* larvae with 10^6^ smooth-type *E. coli* cells were removed from the hemolymph or killed 8 h after inoculation by an unknown immune mechanism (Koizumi et al. 1999a). Phagocytosis may mediate this unknown immune response. Animals have (at least) duplicate safety mechanisms for their physiological functions, which may also be the case for cell-mediated immunity. Therefore, phagocytosis should also play an important role in insect immunity, although this requires further investigation.

Thus, hemocyte aggregates induced to produce by 1 or a few microbial cells (Fig. 1) are thought to play an important role in infection in the natural environment. However, at the same time, the ability of nodules to cope with a large number of invading microorganisms cannot be ruled out as an important survival ability of insects.

## Role of C-type Lectins in the Initiation of Nodule Formation

In the late 1990s, the roles of PAMPs and microorganisms that promote nodule formation began to be elucidated; however, it was not known whether recognition by PRRs occurred in the hemolymph or on the membrane of granule cells (Gillespie et al. 1997). Therefore, roles in nodule formation of major 3 types of PRR present in the hemolymph of insects (i.e., β-glucan recognition protein (βGRP)/Gram-negative bacteria binding protein (GNBP), peptidoglycan recognition protein (PGRP), and C-type lectin) have been investigated.

**Fig. 2.**
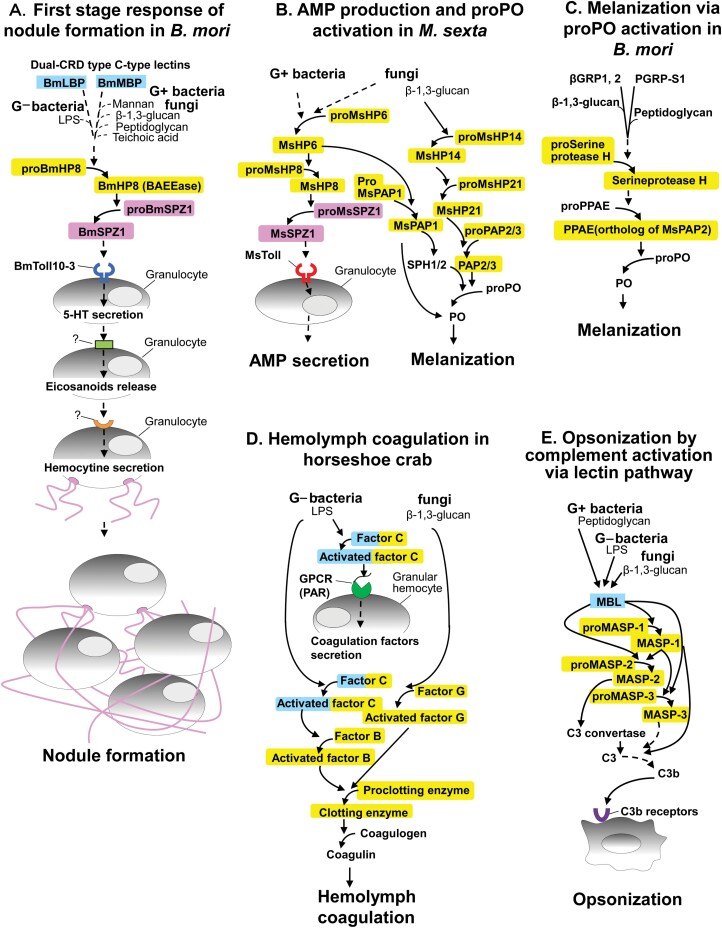
Comparison of the signaling pathways of the first stage of nodule formation in *B. mori* to 4 other immune systems. A) Schematic diagram of the signaling pathways of the first stage of nodule formation in *B. mori*. The diagram includes results from inhibitory experiments that used antisera (Koizumi et al. 1997, 1999a, Watanabe et al. 2006, Tokunaga et al. 2021, Tang et al. 2022) and knockout experiments (Suzuki et al. 2022). The results of inhibitory and knockout experiments were superimposed on the cascading induction scheme for AMP production in *M. sexta* (Kanost and Jiang 2015). The model of the chain reaction of granulocytes to 5-HT and eicosanoids is based on the reports of Kim et al. (2009), Park and Kim (2012), Shestha and Kim (2009), and Shafeeq et al. (2018). B) Schematic diagram of signaling pathways for AMP production and proPO activation in *M. sexta,* as described by Kanost and Jiang (2015). C) Schematic diagram of the signaling pathway for the *B. mori* proPO activation system, which is based on the findings of Ashida and Brey (1998) and Satoh et al. (1999). D) Schematic diagram of the signaling pathways of the hemocyte coagulation system in horseshoe crabs, as described by Kawabata and Muta (2010) and Kawabata et al. (2010) . E) Schematic diagram of the lectin signaling pathway involved in complement activation, as described by Sekine et al. (2012) and Beltrame et al. (2015). The solid and dashed arrows indicate activation based on direct and circumstantial evidence, respectively. Factors highlighted in blue, yellow, and pink are C-type lectin or proteins with a C-type lectin domain, serine proteases or proteins with serine protease domains, and Spätzle, respectively. PO, phenoloxidase; PAP, prophenoloxidase-activating protease; PPAE, prophenoloxidase-activating enzyme; HP, hemolymph proteinase; SPZ, Spätzle; MBL, mannose binding lectin; MASP, MBL-associated serine proteases.

βGRP-1 in *B. mori* larvae was recognized for its ability to activate pro-phenoloxidase (proPO) (Ochiai and Ashida 1988) (Fig. 2). Four types of βGRPs are found in the silkworm genome (Tanaka et al. 2008), while *M. sexta* has 2 types of βGRP: βGRPα and β ( Ma and Kanost 2000, Jiang et al. 2004, Ma et al. 2005, Wang et al. 2011). The ProPO-activating activity of Plodia *interpunctella* is attributed to βGRP (Fabrick et al. 2003). Knock-down assays of 2 GNBPs from pea aphids (*Acyrthosiphon pisum)* reduced phenoloxidase (PO) activity in the hemolymph (Ji et al. 2021). Antisera raised against βGRPs was used to determine that *B. mori* βGRP-2 and -3 were not involved in nodule formation (Tokunaga et al. 2021). These results may imply that βGRPs are not involved in nodule formation in lepidopteran insects.

Overexpression of GNBP-1 in Schneider cells from *Drosophila melanogaster* enhanced AMP production in cells treated with β-1, 3-glucan or lipopolysaccharide (LPS) (Kim et al. 2000). GNBP-3 from *T. molitor* contributed to the activation of proPO and induction of AMP production through activation of the Spätzle processing enzyme (Kan et al. 2008, Yang et al. 2017). A direct role for βGRP/GNBP in nodule formation by lepidopteran insects has not been demonstrated (Fig. 2). However, βGRP/GNBP may activate nodule formation in other insects, specifically dipteran and coleopteran insects, since the signaling pathway for the induction of AMP production and nodule-formation pathway is common in lepidopteran insects, as mentioned in Section 3.

PGRP-S1 and PGRP-S5 from *B. mori*, and PGRP1 from *M. sexta*, were found to be involved in the proPO-activating cascade (Yoshida et al. 1996, Sumathipala and Jiang 2010, Chen et al. 2014) (Fig. 2). PGRP-1 from the coleopteran insect *Holotrichia diomphalia,* and PGRP-LE and PGRP-LC-I from the dipteran insect *D. melanogaster,* were found to have proPO activating properties (Lee et al. 2004, Takehana et al. 2004, Schmidt et al. 2008). The involvement of PGRPs in the induction of AMP production through the Toll pathway in *D. melanogaster* and *Apis mellifera* (Kurata 2014, Wang et al. 2019) implies that PGRPs from dipteran and hymenopteran insects may also play a role in the activation of nodule formation, since the signaling pathway for AMP-production induction and nodule formation is common in lepidopteran insects, as mentioned above.

No reports have indicated that PGRPs are directly involved in nodule formation, or even in the induction of AMP production through the Toll pathway in lepidopteran insects including *M. sexta* and *B. mori* (Fig. 2). Antiserum raised against BmPGRP-S1 did not inhibit nodule-like aggregate formation in vitro (Tokunaga et al. 2021). These findings imply that PGRPs in lepidopteran insects may not be integral to the induction of nodule formation. Hemolymph proteinase 5 (HP5) from *M. sexta* is activated by HP21 and proPO-activating protease 3 (PAP3), which also functions in the proPO activation pathway. Activated HP5 and HP6 both function in the signaling pathway that induces AMP production (Wang et al. 2020). Therefore, in *M. sexta*, HP5 may facilitate crosstalk between the proPO-activating system and the AMP-production-inducing system. PAMP recognition molecules (βGRP/GNBP and PGRP) in lepidopteran insects may also facilitate induction of the first stage of nodule formation, although their role and its significance remain unclear.

Insect C-type lectins are classified into 3 types (Xia et al. 2018) and the production of C-type lectins is up-regulated by microbial stimulation (Rao et al. 2015, Xia et al. 2018). Among these lectins, the activity of the dual-carbohydrate recognition domain (CRD) type C-type lectin, which has no functional domains besides 2 CRDs, has been well studied especially in lepidopteran insects. Recombinant *M. sexta* immulectin (IML)-3 and -4 proteins agglutinated *E. coli*, *Staphylococcus aureus*, and *S. cerevisiae* cells (Yu et al. 2005, 2006). Agglutination may occur when the 2 CRDs on MsIML-3 and -4 join microorganisms together in the hemolymph. The agglutination activity of IMLs may also be unrelated to nodule formation, as the first stage of nodule formation can be induced with a single cell or a few *S. cerevisiae* cells, as mentioned in Section 1.

Nodules are not simple aggregates consisting of microorganisms, but rather aggregated microbial cells and hemocytes (Ratcliffe and Gagen 1977, Sakamoto et al. 2011). During nodule formation, C-type lectin may be relevant to the formation of such complex aggregates. Beads coated with *M. sexta* IML-3 and -4 enhance encapsulation (Yu et al. 2005, 2006). However, encapsulation is a cellular reaction facilitated by plasmatocytes that differs from the first stage of nodule formation, which is mediated by granulocytes. MsIML-2-coated beads were also found to promote phagocytosis in vitro (Ling and Yu 2006). Thus, as PRRs, dual-CRD type C-type lectins may be involved in multiple pathways of cell-mediated immunity.

Two dual-CRD type C-type lectins from *B. mori* are involved in the induction of nodule formation (Fig. 2). LPS-binding proteins (BmLBP) bind to the lipid A part of LPS, which in turn results in binding to various Gram-negative bacteria (Koizumi et al. 1997). Although BmLBP binds well to *E. coli* cells, BmLBP has no direct antibacterial activity against *E. coli* cells (Koizumi et al. 1997). However, anti-BmLBP antiserum markedly inhibits the clearance of *E. coli* cells injected into hemolymph (Koizumi et al. 1997). Glass-adherent cells (granulocytes and plasmatocytes) mixed with plasma and *E. coli* cells, as well as glass-adherent and *E. coli* cells pretreated with plasma, generated nodule-like aggregates in vitro (Koizumi et al. 1999a, Tokunaga et al. 2021). Antiserum against BmLBP significantly suppressed nodule-like aggregate as well as nodule formation in silkworm larvae inoculated with *E. coli* cells (Tokunaga et al. 2021). Two types of Gram-negative bacteria strains that differ in colony appearance exist, namely rough strains and smooth strains. BmLBP bound only to lipid A exposed on the cell surface of rough strains (Koizumi et al. 1999a). *E. coli* cells from rough strains were cleared from the hemolymph of *B. mori* within 30 min, while the smooth strain cells were not cleared until 8 h after injection (Koizumi et al. 1999a). These results imply that BmLBP is a PRR that plays a central role in the induction of nodule formation against Gram-negative bacteria through recognition of lipid A.

*B. mori* multibinding protein (BmMBP) is another dual-CRD type C-type lectin that binds to various types of PAMPs, including peptidoglycan, mannan, and teichoic acid (Watanabe et al. 2006). Antiserum raised against BmMBP suppressed both nodule-like aggregate formation in vitro and the nodule formation induced by *M. luteus* and *S. cerevisiae* cells in vivo (Tokunaga et al. 2021). Therefore, BmMBP is thought to be an important PRR involved in the first stage of nodule formation against Gram-positive bacteria and fungi.

BmLBP and BmMBP are the major C-type lectins in silkworm larval hemolymph. BmIML is also present in the hemolymph, although at very low concentrations (Kim et al. 2003, Takase et al. 2009). It may also function as a PRR during nodule formation, as it is a C-type lectin capable of recognizing a wide range of microorganisms (Kim et al. 2003). Antiserum raised against BmIML also suppressed the first stage of nodule formation which was induced by *M. luteus* and *S. cerevisiae* cells (unpublished data).

In *B. mori* larvae, PRRs for lipoteichoic acid (LTA), β-1, 3-glucan, and LPS exist on what appear to be granulocytes, based on their morphological characteristics (Ohta et al. 2006). Receptors for BmLBP and BmMBP are also thought to be present in these cells. The binding of microorganisms and PAMPs to cells through BmLBP, BmMBP and their putative receptors may play a larger role than direct binding of PRR on cell membranes (Ohta et al. 2006). The binding of microorganisms to granulocytes is thought to be relevant to triggering the first stage of nodule formation and to induce AMP production in the sense of locating the signaling pathway close to granulocytes. Granulocytes located near microorganisms must be activated to initiate the first stage of nodule formation and capture and kill the microorganisms.

Thus, unlike proPO activation, nodule formation and AMP production of the lepidopteran insects is thought to be initiated by the survey of LPS, peptidoglycan, mannan, beta-1, 3-glucan, and teichoic acid by several dual-CRD type C-type lectins in the hemolymph (Fig. 2).

## Serine Proteinase and Spätzle Mediated Signaling Pathway for the First Stage of Nodule Formation

Antiserum raised against *B. mori* hemolymph proteinase 8 (BmHP8), previously referred to as serine protease for benzoyl-arginine ethylester, BAEEase (Jang et al. 2006), suppressed nodule formation in *B. mori* larval hemocoels inoculated with *M. luteus, E. coli*, and *S. cerevisiae* cells (Tokunaga et al. 2021). BmHP8 antiserum also suppressed in vitro nodule-like aggregate formation in mixtures of isolated glass-adherent hemocytes from *B. mori* larvae, plasma, and microbial cells (Tokunaga et al. 2021). Antiserum raised against BmHP14 and BmHP21, which are orthologs of MsHP14 and MsHP21, respectively, and contribute to the proPO-activating serine protease cascade (Wang et al. 2007, Wang and Jiang 2007), did not suppress nodule formation. These results imply that serine proteases not involved in the proPO-activating serine protease cascade function during the first stage of nodule formation (Fig. 2). In addition, antiserum raised against *B. mori* Spätzle1 (BmSPZ1) suppressed both nodule and in vitro nodule-like aggregation (Tang et al. 2022), which indicates involvement of this cytokine in the signaling pathway during nodule formation (Fig. 2).

**Fig. 5.**
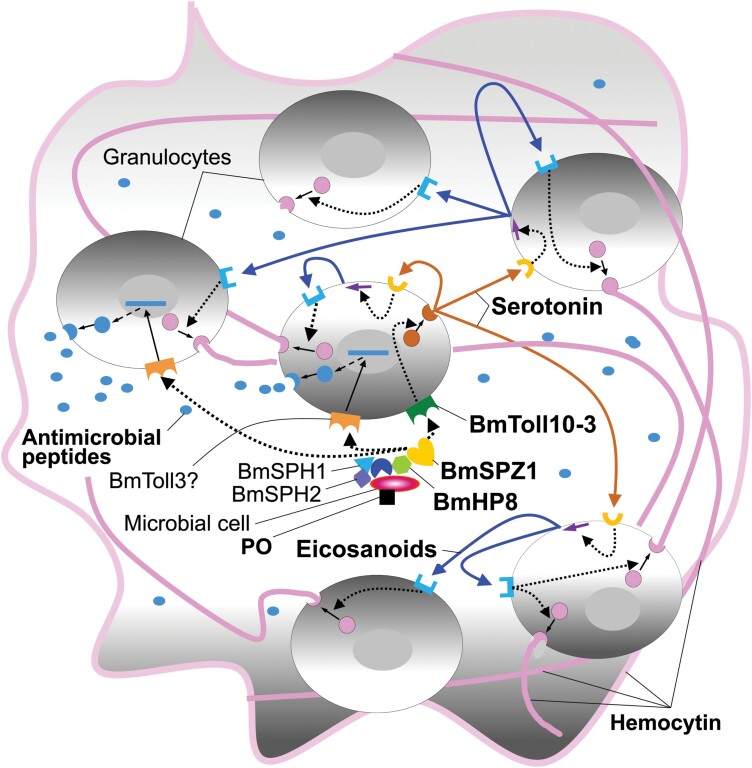
Proposed model for hemocytin-dependent aggregate formation during the first stage of nodule formation in *B. mori.* The model is based on the reports of Kim et al. (2009), Park and Kim (2012), Shestha and Kim (2009), and Shafeeq et al. (2018), which suggested that 5-HT acts upstream in conjunction with eicosanoids to induce nodule formation. The model also takes account of the findings of Tokunaga et al. (2021), Tang et al. (2022), Suzuki et al. (2022), and Otuka and Sato (2023), which imply that BmToll10-3 pathway activation via BmHP8 and BmSPZ1 contributes to aggregation formation in the first stage of nodule formation. Activated BmHP8 on microbial cell(s) is proposed to activate the precursor of BmSPZ1, which in turn activates the BmToll10-3 signaling pathway of the surrounding granulocytes. This process leads to 5-HT secretion, which induces eicosanoid release in an autocrine and/or paracrine manner. Eicosanoids then induce hemocytin secretion in an autocrine and/or paracrine manner, which then forms aggregates composed of hemocytes and microbial cell(s) in the hemolymph. The activation of AMP by BmSPZ1 through a specific BmToll pathway in the aggregate-forming granulocytes was also proposed based on the AMP induction system in *M. sexta* reported by An et al. (2009) and Zhong et al. (2012).

The extracellular signaling cascade that mediates AMP production in *M. sexta* is comprised of MsHP6, MsHP8, and MsSPZ1 (An et al. 2009, 2010) (Fig. 2). MsSPZ1 is involved in the production of attacin-1, cecropin-6, moricin, and lysozyme in hemocytes through activation of the MsToll signaling pathway (Zhong et al. 2012). BmSPZ1 was reported to be associated with the production of attacin-2; cecropin-A1,-B1, and -D1; gloverin-A5 and -B; lebocin-3; and moricin-A1, although hemocyte induction was not confirmed (Wang et al. 2007). Therefore, a signaling pathway consisting of BmHP8 and BmSPZ1 that initiates nodule formation may be homologous to AMP induction in hemocytes. Indeed, hemocytes from the African termite (*Pseudacanthotermes spiniger)* produce the AMP termicin (Lamberty et al. 2001). *Calliphora vicina* hemocytes produce 4 kinds of AMPs (Yakovlev et al. 2017), while *Pseudoplusia includes* produces 2 AMPs, cecropin A and lebocin (Lavine et al. 2005). Hemocytes from *B. mori* produced gloverin-4 (Kaneko et al. 2007), while cultured plasmatocytes and granulocytes produced cecropin (Shimabukuro et al. 1996). These results may imply that BmHP8 and BmSPZ1 are part of a bifunctional signaling pathway in granulocytes that induces AMP production and nodule formation (Fig. 5).

Antiserum raised against BmHP8 inhibited both nodule formation *in B. mori* larvae and nodule-like aggregate formation in vitro followed by treatment with Gram-positive bacteria, Gram-negative bacteria, and fungi, implying that signaling pathways that utilize BmHP8 are common to most microorganisms (Tokunaga et al. 2021) (Fig. 2). As mentioned previously, recognition of inoculated microbial cells by C-type lectins (BmLBP, BmMBP, and BmIML) may regulate activation of a downstream serine protease through a common mechanism (Fig. 2).

**Fig. 3.**
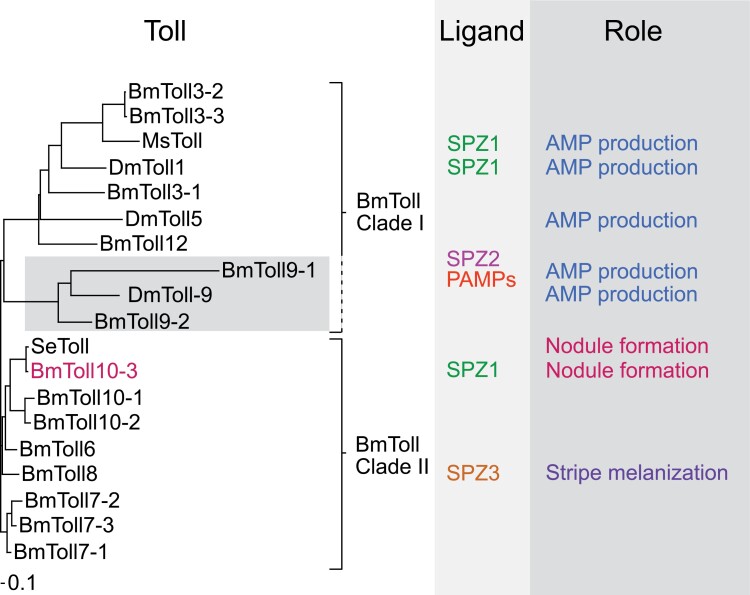
Differentiation of Tolls, as well as their ligands and roles, in *B. mori* and several other insects based on a report by Tanaka et al. (2008). The phylogenetic tree was drawn according to the materials and methods of Suzuki et al. (2022). Information on ligands and their roles were obtained from the following papers: DmToll, Hoffmann (2003); DmToll9, Ooi et al. (2002); DmToll5, Tauszig et al. (2000); BmToll9-1, Yu et al. (2020) and Zhang et al. (2021); MsToll, Ao et al. (2008); BmToll 9-1, Yu et al. (2020); BmToll8, Kondo et al. (2017); and BmToll10-3, Tang et al. (2022) and Suzuki et al. (2022). AMP, antimicrobial peptides; SPZ, Spätzle.

**Fig. 4.**
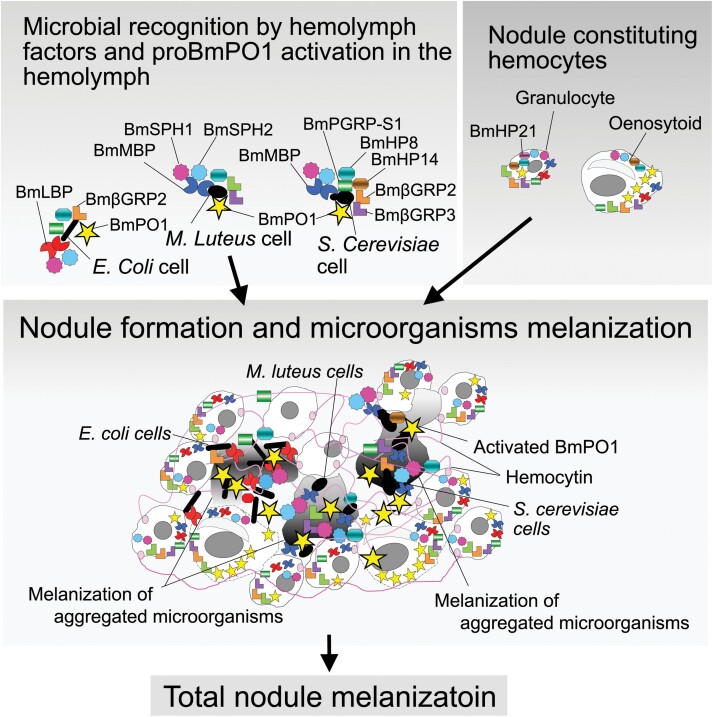
Proposed model for the mode of microorganisms melanization of lepidopteran insect nodules. PO is bound to microbial cells and activated in the hemolymph and then microbial cells are captured by nodules. SPHs bind to microbial cells in the hemolymph through a complex with C-type lectins. The melanization begins in the matrix of the nodule where microbial cells aggregate.

Microorganisms pretreated with *B. mori* larval plasma induce aggregation with hemocytes from *B. mori* larvae in vitro (Koizumi et al. 1999a, Tokunaga et al. 2021). The molecules responsible for nodule formation in granulocytes should also be present on the surface of microorganisms. As mentioned in Section 4, activation of the Toll pathway mediated by BmToll10-3 is thought to trigger nodule formation, which requires BmSPZ1 to be present on, or close to, microbial cells (Figs. 2–4). However, western blot analysis identified BmSPZ1 in hemocytes isolated from hemolymph pretreated with *S. cerevisiae* cells (Tang et al. 2022), but not consistently in *S. cerevisiae* cells pretreated with plasma (unpublished data). In contrast, activated BmHP8 was detected in microorganism cells that were pretreated with *B. mori* larval plasma (Tokunaga et al. 2021). While questions about the detection sensitivity and experimental conditions persist, BmSPZ1 was found not to bind tightly to microbial cells. Continuous diffusion of activated BmSPZ1 from microbial cells can activate more granulocytes around the microbial cells than BmSPZ1 fixed on microbial cells. Further investigation is needed to clarify the process of BmSPZ1 activation. Figure 2 shows a schematic diagram of the serine proteinase cascade that induces the first stage of nodule formation. The results of inhibitory experiments using antisera and *B. mori* larvae were superimposed on the cascading induction scheme for AMP production in *M. sexta* (Kanost and Jiang 2015, Tokunaga et al. 2021, Tang et al. 2022).

Bacterial LPS-induced exocytosis (degranulation) of the sticky protein, coagulogen, from granular hemocytes is key for the horseshoe crab’s innate immunity to infectious microorganisms. The C-type lectin and serine proteinase domains of surveillance proteins against invading microorganisms, including factor C, are involved in the activation of degranulation by granular hemocytes (Ariki et al. 2004) (Fig. 2). The mechanism of blood/hemocyte coagulation in horseshoe crabs has a little similarity to the first stage of nodule formation. An activated G-protein-coupled receptors (PAR) on the cellular membrane are thought to trigger the exocytosis of coagulation factors from granular hemocytes involved in the blood coagulation system of the horseshoe crab (Ariki et al. 2004). PAR was initially considered a candidate for triggering the first stage of nodule formation. However, the PAR molecule triggering the exocytosis of granular hemocytes in the horseshoe crab had not been identified until now. A molecule with high similarity to PARs involved in exocytosis from human platelets has not been identified in the SilkBase *B. mori* gene database (Ma et al. 2005). Indeed, *B. mori* larvae homozygous for the truncated BmToll10-3 allele (BmToll10-3 KO larvae) exhibited almost no nodules in response to an injection of *S*. *cerevisiae* cells (Suzuki et al. 2022). Therefore, PAR is not a major target molecule for the signaling pathway that includes serine protease BmHP8 in *B. mori* larvae.

The serine proteinase domain of factor C, which is a chimeric protein with C-type lectin and serine proteinase domains, is activated upon binding to LPS through the C-type lectin domain in horseshoe crabs (Nakamura et al. 1988). Human mannose-binding lectin (MBL) promotes the autocatalytic properties of MBL-associated serine protease 2 (MASP-2) dimers, and binding of MBL to some PAMPs can enhance this effect (Chen and Wallis 2004). These results imply that the binding of C-type lectin or C-type lectin domain to some PAMPs activates serine proteases and serine protease domains.

MsHP6 functions upstream of the serine protease cascade that induces AMP production in *M. sexta* (An et al. 2009). Therefore, the putative serine protease, BmHP6, in *B. mori* may be the most upstream serine protease in the first stage of nodule formation, and could be activated by BmLBP and BmMBP (Fig. 2). BmHP6 may interact with BmLBP and BmMBP or exist in a complex with BmLBP and BmMBP as well as serine *B. mori* serine proteinase homologs, BmSPH-1 and -2 (Sakamoto et al. 2011, Tokura et al. 2014, Shu et al. 2016). Through activation of BmHP6 by BmLBP or BmMBP on microbial cells, proBmHP8 is activated on microbial cells (Tokunaga et al. 2021). Activated BmHP8 then activates proBmSPZ1, which readily detaches from the microbial cells and binds to granulocytes (Tang et al. 2022), likely through BmToll10-3. Additional research is needed to confirm this mechanism.

In humans, the complement system is activated by 3 pathways: the classical pathway, the lectin pathway, and an alternative pathway; this generates the opsonin, C3b (Alfred et al. 1977). A pathogen coated with C3b binds to complement receptor 1 (CR1) expressed on the surface of phagocytes, which enhances phagocytosis. The lectin pathway includes MBL, which acts as a pattern-recognition molecule for pathogens and activates MBLPs (MASP-1, MASP-2, and MASP-3) (Matsushita 1996, Sekine et al. 2013, Beltrame et al. 2015). The lectin pathway, therefore, involves the serine proteinase cascade comprising MASPs, which is relevant to cell-mediated immunity. This pathway may be evolutionally related to the nodule-formation pathway of insects through C-type lectin, HP6, and HP8. Humoral components and the cellular immune system also cooperate, similar to the first stage of nodule formation.

As discussed above, several dual CRD-type C-type lectins in lepidopteran insects are thought to initiate nodule formation through activation of a serine protease cascade common to AMP production. HP8 binds to invading microorganisms (Tokunaga et al. 2021) and spreads activated SPZ1 (Tang et al. 2022) to transmit information about the presence of microorganisms to surrounding granulocytes in the hemolymph, resulting in the formation of hemocyte aggregates that effectively surround the microorganisms.

## The Toll Pathway Mediated by Toll 10-3 is Associated With the First Stage of Nodule Formation

Each insect species has a different number of Toll family molecules. All 9 family members of human Toll-like receptors (TLRs), orthologs of insect Toll, are involved in various immune responses (Kawai and Akira 2010). Therefore, most Toll family members are expected to play a role in immunity. Indeed, Tanaka et al. (2008) found 14 members of the Toll family in *B*. *mori* (Fig. 3), which were divided into 2 major groups. BmToll3-1, 3-2, 3-3, 9-1, 9-2, and 12 were assigned to clade I and suspected to play roles in AMP production. DmToll1 which is also assigned to clade I induced production of several AMPs, while DmToll5 and DmToll9 induced production of drosomycin in *D. melanogaster* (Tauszig et al. 2000, Ooi et al. 2002) (Fig. 3). MsSPZ1, a possible ligand of Toll in *M. sexta* (MsToll), is involved in the production of attacin-1, cecropin-6, moricin, and lysozyme (An et al. 2009, Zhong et al. 2012) (Fig. 3). The ectodomain of MsToll interacts with MsSpz-C108 (C-terminal active domain of MsSPZ1). Purified recombinant MsSpz-C108 injection activated AMP expression, but this activation was blocked by pre-injection with an antibody against MsToll (Zhong et al. 2012). Real-time PCR indicated that *E. coli*, *S. cerevisiae*, and *Micrococcus lysodeikticus* injection upregulated MsToll in hemocytes (Ao et al. 2008). In addition, MsToll expression was observed in hemocytes that had similar morphological characteristics to granulocytes in *M. sexta* (Ao et al. 2008). These results imply that Tolls in clade I (Fig. 3) are relevant to AMP production via SPZ1 in hemocytes, including granulocytes in *M. sexta*. Granulocytes are involved in the first stage of nodule formation. In addition to melanization (Section 7 and Fig. 4), AMP production is expected to occur in the granulocytes that are involved in nodule formation in lepidopteran insects. BmToll9-1 binds BmSpz2 to promote drosomycin and diptericin gene expression in silkworm larvae (Yu et al. 2020). Expression of BmToll9-1 was confirmed in hemocytes (Wu et al. 2010). However, BmToll9-1 and 9-2 were recently found to be phylogenetically and functionally distant members of Toll clade I (Zhang et al. 2021) (Fig. 3). So, there is some confusion about how BmToll9 functions.

In contrast, BmToll6, 7-1, 7-2, 7-3, 8, 10-1, 10-2, and 10-3 were assigned to clade II (Fig. 3) (Tanaka et al. 2008). These molecules are thought to be involved in the immune system other than AMP biosynthesis (Tanaka et al. 2008). BmToll8 activates signaling pathways in the epidermis that initiate melanin pigmentation to form a striped pattern on the larval body of silkworm laces (Kondo et al. 2017). Although this melanization process is not an immune reaction, the mechanism may have its origins in a primal immune system that depends on the activation of proPO. The BmToll8 signaling pathway that activates melanin pigmentation is still not fully understood.

Knockdown of *S. exigua* myeloid differentiation factor 88 (SeMYD88) or SePelle, which are Toll signaling pathway participants, suppressed nodule formation in *Spodoptera exigua* (Shafeeq et al. 2018). The knockdown of SeToll suppressed nodule formation, implying that the SeToll signaling system is relevant to nodule formation (Park and Kim 2012). However, the results were evaluated 8 h after injection of microorganisms. Therefore, it was unclear whether the inhibition of nodule formation by SeToll knockdown was due to suppression of granulocyte aggregation during the first stage of nodule formation or plasmatocyte adhesion during the second stage. The role of Toll in the first stage of nodule formation was investigated using antiserum against BmToll10-3, an ortholog of SeToll applied in the in vivo nodule formation assay in *B. mori* (Fig. 3). Anti-BmToll10-3 significantly inhibited the first-stage response, while antiserum raised against BmToll3-1, the putative orthologs of DmToll1, did not inhibit the response (Suzuki et al. 2022). When BmToll10-3 was knocked out by genome editing in *B. mori* larvae for the purposes of an in vivo nodule-formation assay, the knockout larvae exhibited no nodules in response to inoculation with 1.0 × 10^7^*S*. *cerevisiae* cells (Suzuki et al. 2022). These results indicate that, of the Toll family members in clade II, BmToll10-3 is involved in the first stage of nodule formation, implying that a reduction of nodules in response to inhibition of the SeToll signaling pathway is also dependent on the first stage of nodule formation. The actual ability of BmSPZ1 to activate the BmToll10-3 signaling pathway is still unclear; however, antiserum raised against BmToll10-3 significantly suppressed the first stage of nodule formation, as did antisera raised against BmHP8 and BmSPZ1 (Tokunaga et al. 2021, Tang et al. 2022, Suzuki et al. 2022). This circumstantial evidence suggests that signaling cascade for AMP induction, which involves C-type lectins, serine proteases, and SPZ1 cross-talk with the BmToll10-3 signaling pathway during the first stage of nodule formation. However, Park and Kim (2012) also showed that the knockdown of SeToll suppresses AMP production in *S. exigua*. Therefore, it should be noted that the role of the BmToll10-3/SeToll signaling pathway, which is expected to activate the nodule formation response, might be still poorly understood.

In humans, TLR 4 triggers the formation of aggregates within a network structure, referred to as neutrophil extracellular traps (NETs) (Chen et al. 2021). These aggregates consist of neutrophiles, platelets, and microorganisms that kill bacteria, viruses, and fungi (Urban et al. 2006, Narasaraju et al. 2011). Although the evolutionary relationship between nodule formation and NETs is not known, it is clear that Toll and TLR are involved in the cell-mediated immunity that leads to the formation of cell aggregates.

These findings suggest that, in lepidopteran insects, SPZ1 transmits a signal to granulocytes via the Toll 10-3 ortholog to induce nodule formation, a cell-mediated immune response. On the other hand, whether Toll 10-3 ortholog or Toll 3 ortholog is involved in induction of antimicrobial peptide production in hemocytes is still confused. However, SPZ1 is thought to simultaneously induce both nodule formation and AMP production in hemocytes.

## Eicosanoids and Serotonin Trigger Hemocyte Aggregation

Eicosanoids are C20 polyunsaturated fatty acids that mediate immune responses in insects (Stanley and Kim 2014). In *M*. *sexta, G. mellonella*, *Agrotis ipsilon, S. exigua, Ostrinia nubilalis*, and *Gryllus assimilis* larvae, nodule formation is strongly suppressed by inhibitors of the eicosanoid biosynthetic pathway (Miller et al. 1994, 1999, Jurenka et al. 1997, Mandato et al. 1997, Tunaz et al. 2003, Shestha and Kim 2009). Knockout of the prostaglandin E2 (PGE2) eicosanoid receptor suppressed nodule formation in *S*. *exigua* larvae (Kim et al. 2020). Nodule formation was also inhibited by infection with *Xenorhabdus nematophilus*, which suppressed phospholipase A2-dependent eicosanoid production in *M. sexta* (Park et al. 2003, Kim et al. 2005). These results imply that eicosanoids are involved in the induction of nodule formation. However, the results were obtained 1–12 h after injection of the microorganisms, which almost overlaps the second stage of nodule formation. The first stage of nodule formation in *B. mori* occurs within 30 s of microorganism invasion (Arai et al. 2013), while the second stage involves plasmatocyte adhesion to the nodule, which affects the response rate and was initiated 2–6 h after injection with *G. mellonela* and *P. brassicae* microorganisms (Ratcliffe and Gagen 1977). These experiments were conducted with *S*. *exigua* and did not show whether eicosanoids were involved in the first stage response. Experiments involving *B. mori* were used to address this knowledge gap. Eicosanoid biosynthesis inhibitors (the arachidonic acid inhibitor dexamethasone and PGE2 inhibitor diclofenac sodium) significantly suppressed nodule formation within 1 min of injection with 2.0 × 10^5^*S. cerevisiae*. In addition, PGE2 itself induced nodule-like aggregate formation (Otuka and Sato 2023). The aggregates induced by PGE2 comprised hemocytes tethered together by hemocytin and had structural characteristics similar to nodules (Otuka and Sato 2023). These findings imply that eicosanoid release is involved in the first stage of nodule formation in lepidopteran insects (Figs. 1 and 2).

Serotonin (5-hydroxytryptamine; 5-HT) is a neurotransmitter and hormone biosynthesized from tryptophan. In humans, it has multiple physiological functions, including signal transmission in thrombopoiesis. In *S. exigua*, 5-HT antagonists, Fenclonine suppressed nodule formation, whereas 5-HT rescued it, implying that 5-HT is involved in nodule formation following *E. coli* injection; however, these findings were obtained 4–8 h after injection (Kim et al. 2009). Fenclonine significantly inhibited nodule formation within 1 min in *B. mori* following injection with 2.0 × 10^5^*S. cerevisiae* (Otuka and Sato 2023). Moreover, 5-HT alone induced the formation of nodule-like aggregates made of hemocytes tethered together by hemocytin (Otuka and Sato 2023). In addition to eicosanoids’ release, 5-HT secretion is now considered to be involved in the first stage of nodule formation (Fig. 2). Notably, both U73122, an inhibitor of Gαq, and pertussis toxin, an inhibitor of Gαi, inhibited nodule formation almost completely in *B*. *mori* larvae (Suzuki et al. 2011). In mammalian cells, G-protein-coupled receptor (GPCR) in combination with Gαq or Gαi function as a receptor for eicosanoid and 5-HT (Masson et al. 2012, Moreno 2017).

The 5-HT-induced-nodule-like aggregate formation is inhibited by dexamethasone, an eicosanoid biosynthesis antagonist, indicating that 5-HT acts upstream of eicosanoid release (Kim et al. 2009). In addition, as above mentioned, nodule formation was under control of GPCR/phospholipase A2-dependent eicosanoid production in *M. sexta* (Park et al. 2003, Kim et al. 2005). These may imply that the Toll signaling pathway regulates 5-HT secretion rather than eicosanoid biosynthesis (Shestha and Kim 2009, Park and Kim 2012, Shafeeq et al. 2018) (Figs. 2 and 5). However, it is still uncertain whether Toll signaling pathway directly regulates 5-HT secretion in a single granulocyte. Further studies are needed to elucidate the full effect of the Toll signaling pathway on granulocytes.

As described above, although the order of 5HT and eicosanoid release is still unclear, it is thought that granulocytes release hemocytin and aggregate through autocrine or paracrine reactions induced by 5HT and eicosanoids.

## Role of Hemocytin in Hemocyte Agglutination

The first stage of nodule formation was found to be dependent on an adhesive protein that is exocytosed by granulocytes (Ratcliffe and Gagen 1977, Arai et al. 2013). Hemocytin is a sticky protein of *B. mori* that promotes aggregation of hemocytes (Kotani et al. 1995) and was found in the granules of granulocytes and substances discharged from hemocytin-containing granules when granulocytes were exposed to air on a glass slide (Arai et al. 2013). The substances formed an intercellular network structure with hemocytes aggregated through the sticky threads (Arai et al. 2013). Nodules observed on the glass slide smears showed hemocytes and microorganisms buried in the exocytosed substances from hemocytin-containing granules (Arai et al. 2013). These results imply that hemocytes and microorganisms were trapped by the sticky network structure formed by hemocytin in *B. mori* (Fig. 5). Indeed, hemocytin may be associated with agglutination of *Nosema bombycis* cells and hemocytes in the hemolymph of *B. mori* larvae. Knockdown assays of hemocytin resulted in increased *N. bombycis* proliferation within the insect (Ni et al. 2020). Hemocytin is a large multi-domain protein considered to be an ortholog of human von Willebrand factor (Kotani et al. 1995), which is involved in platelet aggregation and has several coagulation-associated domains, such as coagulation factor VIII of the human (Toole et al. 1984, Jenny et al. 1987). Nodule formation and platelet aggregation may be evolutionarily related.

The ortholog of hemocytin in *D. melanogaster* is referred to as hemolectin. Hemolectin is a major constituent of clot proteins, and is specifically expressed in embryonic and larval hemocytes, including plasmatocytes (Goto et al. 2003, Scherfer et al. 2004). After pricking, the amount of hemolymph bleeding was much higher in hemolectin knockdown *D. melanogaster* larvae compared to control larvae, which implies that hemolectin is involved in clotting and injury repair in *D. melanogaster* (Goto et al. 2003). Hemolymph taken from genetically modified *D. melanogaster* with hemolectin deficiencies did not induce bacterial aggregation (Lesch et al. 2007). Furthermore, hemolectin*-*deficient *D. melanogaster* larvae were more susceptible to a mutant strain of *Serratia marcescens* (Chang et al. 2012). These findings indicate that hemolectin may be a major factor in the clotting system of *D. melanogaster*. The clotting system of *D. melanogaster* is similar to the nodule-formation system in terms of the ability to aggregate hemocytes and microorganisms (Bidla et al. 2005, Theopold et al. 2014). Although they represent circumstantial and indirect evidence, these results may imply indirectly that hemocytin is a major component of the sticky compound produced by nodules. Additional research is needed to understand the function of hemocytin fully, as well as the mechanisms of hemocyte and microorganism aggregation.

Tiggrin and Fondue have been reported to be clot constituents in *D. melanogaster*. However, neither was found by a Blast search of a lepidopteran insect database (Scherfer et al. 2004, Lindgren et al. 2008). In contrast, RNA interference (RNAi) of Noduler reduced the number of nodules generated in response to microbial injection (Gandhe et al. 2007). RNAi of *B. mori* Nodular (BmReeler 1), also suppressed nodule formation in response to bacterial injection, while recombinant *B. mori* Nodular protein injection rescued nodule formation in knockdown larvae (Bao, et al. 2011). Nodular, a Reeler domain-containing protein, has been reported to be a sticky protein that binds LPS, lipotechoic acid, and beta-1, 3 glucan components of microbial cell walls with hemocytes in the Indian saturniid silkmoth (*Antheraea mylitta)*. Therefore, Nodular was thought to be an aggregation-forming protein that function in the first stage of nodule formation (Gandhe et al. 2007). However, western blotting of the normal plasma and hemocytes from *A. mylitta* larvae detected only low levels and almost no Noduler respectively (Bao et al. 2011). In the same way, *B. mori* Noduler in the hemolymph can bind microbial cells, but western blotting of the nodules formed by inoculation with microbial cells to *B. mori* larvae and of hemocytes detected almost no *B. mori* Noduler (unpublished data of the author). Also, anti-Noduler antiserum could not detect Noduler protein in the sections of nodules induced by microbial inoculation in *B. mori* larvae, although anti-hemocytin antiserum detected strong signals. These suggested that Noduler is not accumulated in the hemocyte and secreted from the hemocytes like hemocytin. Further studies are required to understand where and when Noduler functions in association with the first stage of nodule formation.

As described above, it is necessary to further investigate how factors other than hemocytin are involved in the formation of granulocyte aggregates. However, the lepidopteran orthologs of the von Willebrand factor, which is a major factor in human platelet aggregation, seem to play an important role in the formation of nodules, including the trapping of microorganisms.

## Mechanisms of Localization of PO and proPO-Activating Factors to the Nodule

Factors involved in the melanization of hemolymph and the mechanisms of activation have been thoroughly investigated in *M. sexta* (Kanost and Jiang 2015) (Fig. 2). Some of the orthologous factors in *B. mori* larvae are also involved in melanization (Fig. 2). PRRs, including BmβGRP and BmPGRP, hemolymph proteinases (serine protease H and S in *B. mori*), and BmproPO-activating protease (PAP; also known as proPO-activating enzyme: PPAE) were found to be involved in melanization (Ashida and Brey 1998) (Fig. 2).

Melanization of nodules was observed in tissue sections of *G. mellonella*; it started 5–30 min after injection of the microbial cells, primarily in the area of the matrix of the nodule (Ratcliffe and Gagen 1977). In *B. mori*, melanization started around 30 min after injection in the inter-hemocyte space of the nodule, which consists of *E. coli* cells and matrix (Sakamoto et al. 2011). The melanization of nodules is thought to begin within the aggregated bacteria and to eventually extend to the surrounding hemocytes. Nodule melanization in lepidopteran insects produces hard aggregates that are thought to prevent the escape of microorganisms. In larval *Drosophila*, PO is not required for the formation of the initial soft clot, but is necessary for cross-linking of the soft clot to form a hard clot (Lindgren et al. 2008).

The C-type lectins, MsIML-1, -2, and -4 of *M. sexta*, promote the melanization of beads coated with larval plasma. MsIML2 has been proposed to bind SPH-1 and -2, PAP, and proPO, which are factors involved in melanization (Yu et al. 2003). In *B. mori,* precursor forms of BmSPH-1 and -2 were complexed with BmLBP or BmMBP in the hemolymph and localized on *E. coli*, *M. luteus*, and *S. cerevisiae* cells through BmLBP or BmMBP, and eventually BmSPH-1 and -2 were activated on microbial cells (Sakamoto et al. 2011, Tokura et al. 2014, Shu et al. 2016) (Fig. 4). Melanization begins within the microbial aggregates (matrix part) of the nodule. Immune histochemical examination showed that BmSPH-1, BmSPH-2, BmLBP, BmMBP, and BmPO were concentrated with aggregated microbial cells in the nodule (Sakamoto et al. 2011, Tokura et al. 2014) (Fig. 4). These findings indicate that PO-activating factors localized in the nodule through C-type lectins, which is important for nodule melanization. Western blot analysis detected ProPO from microbial cells pre-incubated with *B. mori* larval plasma, which implies that proPO is activated on microbial cells in the hemolymph (Tokura et al. 2014, Shu et al. 2016). Furthermore, western blot analysis revealed higher levels of the precursor and activated forms of BmHP14 and BmHP21 in microorganism-induced nodules compared to free hemocytes, implying that they are concentrated into nodules from the plasma (Shu et al. 2016). Two serine protease homologs (BmSPH1 and BmSPH2), 2 serine proteases (BmHP14 and 21), and proPO may be activated on microbial cell(s) in the hemolymph. Activated PO then begins the melanization process on the microorganisms in the nodule (Fig. 4). βGRP and PGRP are PRRs that trigger proPO-activating serine protease cascade (Ashida and Brey 1998). One role of C-type lectins, including BmLBP, BmMBP, and MsIML2, may be to concentrate several melanization factors, including SPH-1 and SPH-2, on microbial cells. This process would be important for efficient and place-restricted melanization of microorganisms trapped in the nodule, as well as for the suppression of hemolymph melanization (Sakamoto et al. 2011), which could harm insect tissues.

Granulocytes are the primary type of hemocytes found in nodules, although a few oenocytoids are involved in *B. mori* (Arai et al. 2013). Immunohistological analysis of granulocytes and oenocytoids revealed the accumulation of large amounts of PRRs, such as BmPGRP-S1, BmβGRP1, BmβGRP2, and BmβGRP3, which are thought to be involved in the activation of proPO. Western blot analysis indicated that serine proteinases (BmHP14 and BmHP21), as well as SPHs (BmSPH-1 and BmSPH-2), also accumulated in granulocytes and oenocytoids (Shu et al. 2016). These factors may be involved in the melanization of hemocytes in the nodule, although their function and activation mechanism are unknown (Fig. 4).

The melanization of nodules is thought to progress through the steps shown in Fig. 2, as the mode of melanization of the hemolymph (Ashida and Brey 1998, Satoh et al. 1999, Kanost and Jiang 2015). ProPO appears to be activated on microbial cells in the plasma of lepidopteran insects (Yu et al. 2003, Sakamoto et al. 2011, Tokura et al. 2014). They were found to be covered by aggregated granulocytes and a layer of hemocytin during the first stage of nodule formation (Arai et al. 2013, Otuka and Sato 2023) (Fig. 4). ProPO may be primarily activated on microbial cells, and the melanization of nodules occurs in the closed environment of the nodule (Fig. 4). In fact, when microorganism cells were injected into *B. mori* larvae, the melanization of the hemolymph hardly progressed; only the nodules were more melanized (Sakamoto et al. 2011).

During melanization, reactive oxygen species (ROS) and reactive nitrogen species (RNS) are produced (Nappi et al. 2009), which likely significantly contribute to the killing of invading microorganisms (Molina-Cruz et al. 2008). However, ROS and RNS also have cytotoxic activity against insect ‘self’ tissues (Ha et al. 2005). Therefore, it is necessary for insects to have a mechanism that efficiently melanizes microorganisms that have invaded the hemocoel, while also minimizing the melanization of tissues. Kanost’s group elucidated the mechanism of melanization of the hemolymph, and argued for the need for a mechanism to realize these 2 complex and contrasting events (Yu et al. 2003). Therefore, the authors considered the proposed melanization system in the nodule to be extremely reasonable in the sense of effective and safe killing of invading microorganisms.

## Conclusion

The first stage of nodule formation begins within 1 min of microbial inoculation, when hemocytes form aggregates (Ratcliffe and Rowley 1979, Arai et al. 2013, Otsuka et al. 2023). Phagocytosis is observed much later in the process; the speed of the reaction implies that nodule formation is an important immune response (Ratcliffe and Rowley 1979). The typical nodules attached to organs may result from the coalescence of small hemocyte aggregates induced by inoculation with millions of microbial cells. Therefore, the observed nodules may be artifacts of inoculation with an unnatural quantity of microbial cells. In contrast, many hemocyte aggregates in the hemolymph contain only 1 captured microbial cell, which implies that hemocyte aggregate formation can be triggered even by only 1 microbial cell (Otsuka et al. 2023) (Fig. 1). Thus, system of nodule formation should have an important role as a natural response in the invasion of a single cell or small numbers of cells of a microorganism.

The first stage of nodule formation in *B. mori* larvae is thought to be initiated by C-type lectins in the hemolymph (Koizumi et al. 1997, 1999a, 1999b, Tokunaga et al. 2021) (Fig. 2). C-type lectins may activate a serine proteinase cascade that affects both AMP production and the first stage of nodule formation (Fig. 2). Activated BmHP8 binds to microbial cells, which implies that BmHP8-bound microbial cells can trigger hemocyte aggregation (Tokunaga et al. 2021) and also trigger AMP production against the aggregated hemocytes (Fig. 5).

To produce meaningful results under natural conditions, the first stage of nodule formation must be studied in terms of its ability to respond to a single microorganism. The detection of activated BmSPZ1 on granulocytes implies that it may be activated by BmHP8 on the microorganism (Tang et al. 2022). BmToll10-3 located on neighboring granulocytes is activated by the binding of activated BmSPZ1, which activates the Toll10-3 signaling pathway (Suzuki et al. 2022) (Fig. 5). Thus, numerous hemolymph proteins, including C-type lectins, serine proteases, and a cytokine (Spätzle), are involved in the induction of a cell-mediated immune system, the first stage of nodule formation (Fig. 2).

In *B. mori* larvae, both 5-HT and PGE2 injection induced nodule-like aggregates in the hemolymph within 1 min (Otuka and Sato 2023). In *S. exigua* larvae, nodule formation rescued by 5-HT inoculation was inhibited by dexamethasone (Kim et al. 2009). Therefore, activation of the Toll signaling pathway is thought to trigger a two-step process of 5-HT and eicosanoid secretion and release (Otuka and Sato 2023) (Figs. 2 and 5). The secretion of a viscous compound was reported to be associated with the first stage of nodule formation (Ratcliffe and Rowley 1979). The nodule-like aggregates induced by 5-HT and eicosanoids were buried in hemocytin (Arai et al. 2013, Otuka and Sato 2023). Therefore, the first stage of nodule formation may begin with secretion of a viscous protein (hemocytin) in response to eicosanoids (Fig. 5).

The orthologs of many signaling factors involved in *M. sexta* proPO activation have also been found in the nodules of *B. mori* larvae (Sakamoto et al. 2011, Tokura et al. 2014, Kanost and Jiang 2015, Shu et al. 2016) (Fig. 4). SPHs, and PAP have been found to bind to microbial cells in the hemolymph through a complex with C-type lectin, while precursor and activated PO have been isolated from bacteria pretreated with hemolymph (Yu et al. 2003, Sakamoto et al. 2011, Tokura et al. 2014, Shu et al. 2016). Therefore, microorganism melanization initiation in the nodule should depend on PO, which is activated in the hemolymph and binds microbial cells (Ratcliffe and Rowley 1979, Sakamoto et al. 2011) (Fig. 4). Areas of aggregated hemocytes in the nodule experience delayed melanization (Ratcliffe and Rowley 1979, Sakamoto et al. 2011) (Fig. 4). Nodule-forming hemocytes, granulocytes, and oenocytoids produce and store most of the melanization-inducing factors, including proPO (Shu et al. 2016) (Fig. 4). These factors may be important for the melanization of aggregated hemocytes in the nodule (Tokura et al. 2014). Thus, 2 antimicrobial systems, namely AMP production and melanization are coordinated in the nodule (Figs. 2, 4, and 5). As previously described, in lepidopteran insects, the cell-agglutination reaction’s rapidity, response potential, and cooperation with the sterilization system in the first stage of nodule formation imply that it may play a major role in preventing the invasion and proliferation of microbial cells.
